# Recipient vessels for free flaps in advanced facial oncologic defects

**DOI:** 10.1016/j.bjorl.2023.03.008

**Published:** 2023-04-06

**Authors:** Bruno Albuquerque Sousa, Fernando Luiz Dias, Marcus A. Acioly de Sousa, Marco Antônio Pinto, Jéssica Marquet Silva, Cláudio Roberto Cernea

**Affiliations:** a Instituto Nacional do Câncer Brasileiro, Departamento de Cirurgia de Cabeça e Pescoço, Rio de Janeiro, RJ, Brazil; b Universidade Federal do Rio de Janeiro, Departamento de Neurocirurgia, Rio de Janeiro, RJ, Brazil; c Universidade São Paulo, Departamento de Cirurgia de Cabeça e Pescoço, São Paulo, SP, Brazil

**Keywords:** Free tissue flaps, Head neck cancer, Microanastomosis, Superficial temporal vessels, Cervical vessels

## Abstract

**Objectives:**

To prospectively compare the results of microvascular flap reconstruction of midface and scalp advanced oncologic defects using superficial temporal versus cervical as recipient vessels.

**Methods:**

This is a parallel group clinical trial with 1:1 allocation ratio of patients who underwent midface and scalp oncologic reconstruction with free tissue flap from April 2018 to April 2022 in a tertiary oncologic center. Two groups were analyzed: those in whom superficial temporal vessels were used as the recipient vessels (Group A) and those in whom cervical vessels were used as the recipient vessels (Group B). Patient gender and age, cause and localization of the defect, flap choice for reconstruction, recipient vessels, intraoperative outcome, postoperative course, and complications were recorded and analyzed. A Fisher’s exact test was used to compare outcomes between the 2 groups.

**Results:**

On the basis of the different recipient vessels, 32 patients were randomized into 2 groups, and of these 27 patients completed the study: Group A with superficial temporal recipient vessels (n = 12) and Group B with cervical recipient vessels (n = 15). There were 18 male and 09 female patients with an average age of 53.92 ± 17.49 years. The overall flap survival rate was 88.89%. The overall complication rate for vascular anastomosis was 14.81%. The total flap loss rate in patients with superficial temporal recipient vessels was higher than the complication rate in those with cervical recipient vessels but with no statistical significance (16.67% vs. 6.66%, *p* = 0.569). Minor complications occurred in 05 patients without statistical significance between the groups (*p* = 0.342).

**Conclusion:**

In the group with superficial temporal recipient vessels, the postoperative rate of free flap complications was similar than the cervical recipient vessel group. Therefore the use of superficial temporal recipient vessels for midface and scalp oncologic reconstruction could be a reliable option.

## Highlights

• Advanced oncologic defects of midface and scalp are challenging for reconstruction.
• Free ﬂaps in the midface and scalp region are the gold standard for advanced cases.
• No consensus in the literature on the best recipient vessels for microanastomosis.
• Superficial temporal and cervical vessels are reliable options for microanastomosis.

## Introduction

Advanced oncologic defects of the midface and scalp are a signiﬁcant challenge to the reconstructive head and neck surgeon, who must consider the need for midfacial projection, rehabilitation, and function restoration. Soft free tissue flaps, such as anterolateral thigh flaps, *latissimus dorsi* myocutaneous flaps and rectus abdominis flaps are excellent reconstructive options for advanced cases, adequately repairing large defects and filling dead spaces.^1^, ^2^, ^3^, ^4^, ^5^ One important aspect of using free tissue ﬂaps in the midface and scalp region is the choice of the recipient vessels for the microanastomosis. Cervical vessels are often the first option in head and neck reconstruction, especially when the lower third of the face is involved. However, if recipient vessels in the neck are selected for midface and scalp reconstruction, a long vascular pedicle must be harvested. In addition, the surgical team should take into consideration the history of previous neck dissection or prior radiotherapy, which can compromise the outcome of microanastomosis. For these cases, the superﬁcial temporal vessels could be used as a recipient target, due to their ideal location and straightforward dissection.^6^, ^7^, ^8^, ^9^

The superﬁcial temporal vessels have been shown to have comparable diameters to the cervical vessels, with increased proximity to defects in the midface and scalp. Yet, because of their variable diameter and tortuous anatomy, these vessels have been considered to be at risk for postoperative vasospasm and thrombosis, and its use has been restricted to selected cases.^10^, ^11^ The aims of this study are to demonstrate a prospective study in midface and scalp advanced oncologic reconstruction using free tissue flap and to compare the postoperative outcomes based on superficial temporal versus cervical recipient vessels.

## Methods

We performed a parallel group clinical trial in patients who underwent midface and scalp reconstruction with free tissue flaps in the department of head and neck surgery of the Brazilian National Cancer Institute from April 2018 to April 2022. This study was approved by the IRB of the Brazilian National Cancer Institute and registered in an international clinical trials database (ClinicalTrials.gov identifier: NCT05749120). All participants signed an informed consent form. The following inclusion criteria were employed: (1) Patients eligible to subtotal, total, or extensive radical oncologic maxillectomy, or advanced scalp oncologic resection; (2) Patients with clinical conditions suitable for major surgery; (3) Complete medical records. Patients were randomized into two groups by the main author in blocks of four patients for each technique (one to one ratio) who were blinded to the intervention, namely Group A: the superficial temporal recipient vessels; and Group B: the cervical recipient vessels. Patient gender and age, cause and classification of the defect, flap choice for reconstruction, recipient vessels, postoperative course, and complications were recorded and analyzed. Patients who died within a period of less than 30 days of the surgical intervention or who missed follow-up for a period of less than 3 months were excluded.

### Statistical analysis

Sample-size estimation was performed using OpenEpi (openepi.com). In Brazil, the estimated incidence of non-melanoma advanced skin cancers that affect the midface and scalp and are eligible for surgery is 800 cases/year (see https://www.gov.br/inca/pt-br/assuntos/cancer/numeros/estimativa consulted in 02/18/2023). We estimated a sample-size of 26 patients with 95% confidence level. Considering an attrition rate of 20%, we set the sample-size at 32 patients. A Fisher’s exact test was used to compare outcomes between the 2 groups. The *p* < 0.05 was considered statistically significant.

## Results

**Fig. 1:**
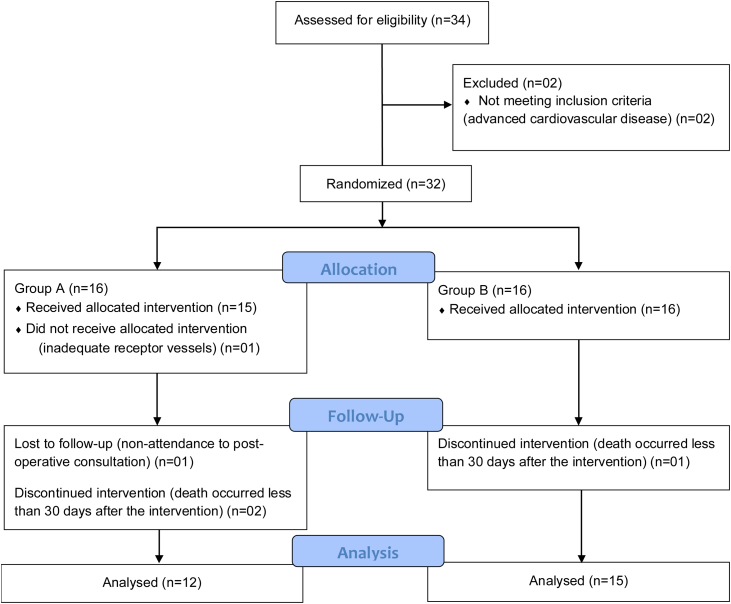
Study design.

**Fig. 2:**
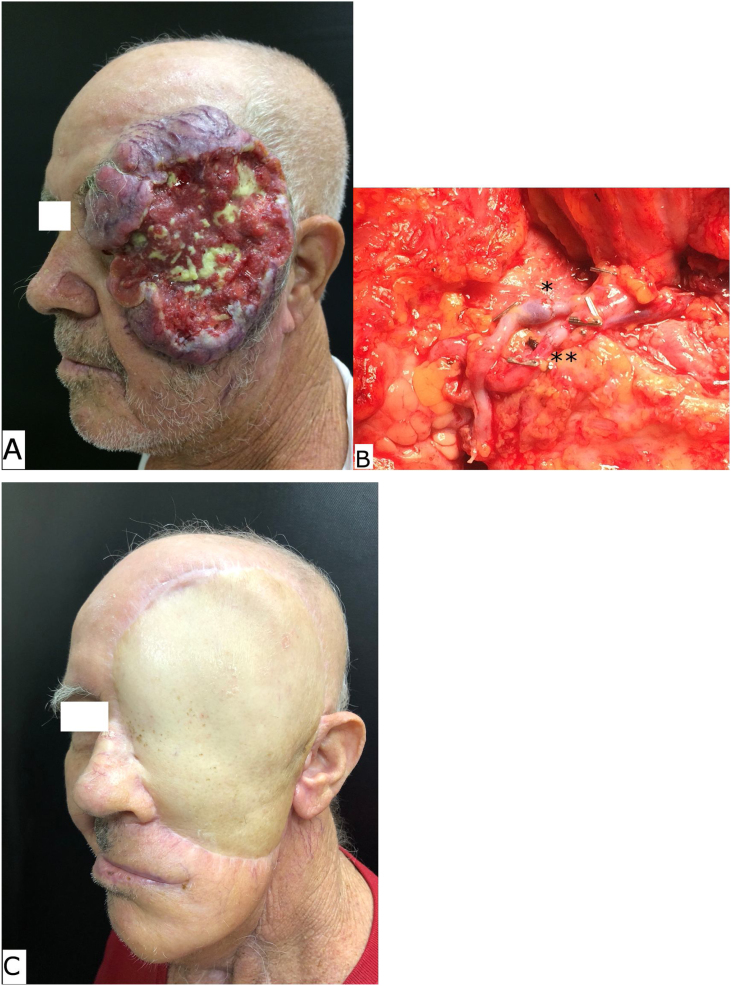
(A) Pre-operative aspect of an advanced squamous cell carcinoma of the left midface. (B) The cervical recipient vessels: single asterisk demonstrates the microanastomosis of facial vein with the flap vein and double asterisk demonstrates the microanastomosis of facial artery with the flap artery. (C) The late post-operative aspect of an anterolateral thigh free flap inserted in the surgical site.

**Fig. 3:**
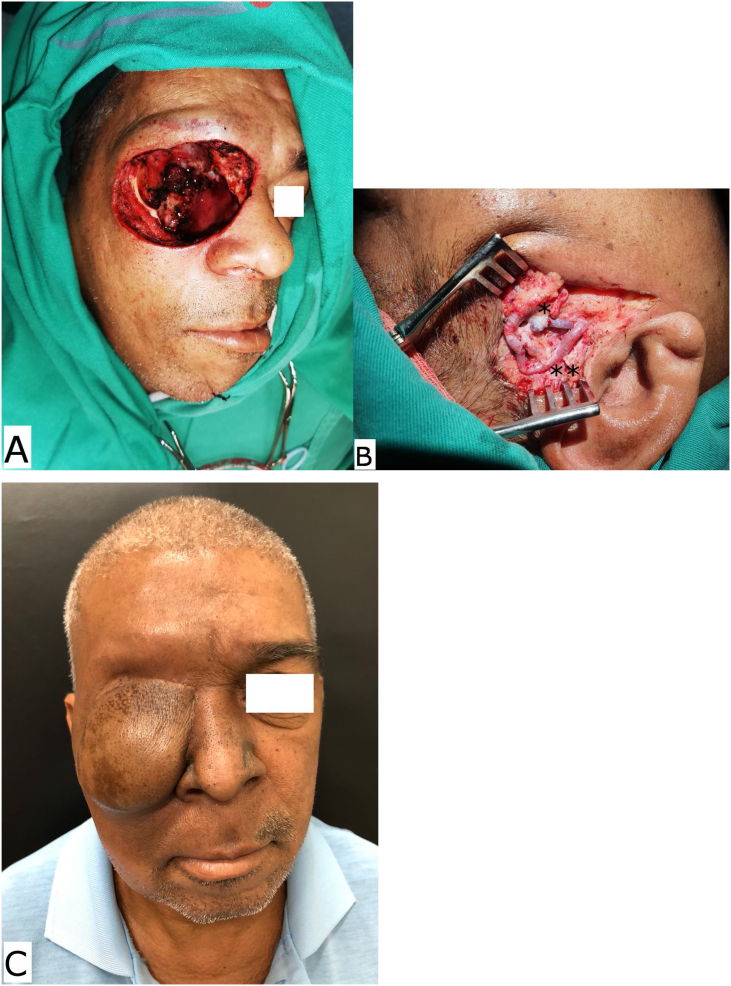
(A) Per-operative aspect of an extended right orbit exenteration due an advanced cutaneous squamous cell carcinoma. (B) The superficial temporal recipient vessels demonstrate the microanastomosis of the superficial temporal vein with the flap vein (single asterisk) and the double asterisk demonstrates the microanastomosis of the superficial temporal artery with the flap artery. (C) The late post-operative aspect of an anterolateral thigh free flap inserted in the right midface.

Thirty-two patients were selected and randomized into two groups. Five patients were excluded from the study. The total study period was developed prospectively over 4 years (from April 2018 to April 2022) with the recruitment period occurring during the initial 45 months. The minimum follow-up of the patients was 3 months, enough time to assess major and minor complications. The diagram describes the participants flow (Fig. 1). Twenty-seven patients were analyzed: Group A with superficial temporal recipient vessels (n = 12) and Group B with cervical recipient vessels (n = 15). There were 18 male and nine female patients, with an average age of 53.92 ± 17.49 years, equally distributed among groups (*p* = 0.448 e *p* = 0.588, respectively). Twenty-three patients underwent immediate reconstruction after ablative tumor surgery whereas four, all disease-free, underwent secondary reconstruction. Squamous cell carcinoma was the most prevalent histology in both groups (*p* = 0.638). The midface was the most affected primary site, with no statistical difference between groups A and B (*p* = 0.294). Of the 27 patients, 85.18% had anterior skull base involvement, without statistical difference between the groups (*p* = 0.130). Patient’s characteristics of group A and B are listed on Table 1. Fig. 2, Fig. 3 demonstrate the intraoperative and postoperative surgical aspects.

**Table 1:** Patients characteristics and type of free tissue flap.

	Group A	Group B	
	Superficial temporal recipient vessels (n = 12)	Cervical recipient vessels (n = 15)	*p*-value
Gender (Female / Male)	05 / 07	04 / 11	0.448
Age (mean ± SD)	51.83 ± 16.62	55.60 ± 18.57	0.588
Histology			
Squamous cell carcinoma	05	08	0.638
Basal cell carcinoma	03	02	
Sarcoma	01	04	
Others	03	01	
Primary site			
Scalp	03	01	0.294
Midface	09	14	
Anterior skull base involvement	05	11	0.130
Free tissue flap			
ALTF	06	07	‒
RAF	01	08	
RFF	03	‒	
LDF	02	‒	

ALTF, Anterolateral Thigh Flap; RAF, *Rectus Abdominis* Flaps; LDF, L*atissimus Dorsi* myocutaneous Flaps; RFF, Radial Forearm Flap.

In Group B, the following recipient arteries were used: facial artery (n = 9), superior thyroid artery (n = 4), and lingual artery (n = 2). The recipient cervical veins used in this group were: facial vein (n = 11) and internal jugular vein (n = 4). Vein grafts were not used. All microanastomosis were performed in an end-to-end fashion except for four cases, in which the anastomosis with the internal jugular vein were performed using an end-to-side technique. Only one patient in Group B required early return to the operating room due to microvascular vascular anastomosis obstruction, with successful resolution. Arterial thrombosis developed in one patient in Group B on the 2nd postoperative day, with total flaps loss. Two patients in Group A suffered total flap loss: one due to venous thrombosis on the 3rd postoperative day, and one due to arterial thrombosis on the 5th postoperative day. Both patients underwent a late successful reconstruction with another free tissue flap. No other major complications occurred in this group.

In the present series, the overall flap survival rate was 88.89% (24 of 27 flaps). All major complications involved microvascular thrombosis, with a complication rate of 14.81% (4 of 27 patients). Of the three patients who developed total flap loss, two were in Group A (16.67%) and one was in Group B (6.66%), not significant (*p* = 0.56). Minor complications such as seroma, partial dehiscence or infection occurred in 5 patients (18.51%). There was no statistically significant difference in the comparison between groups (*p* = 0.342). The vascular pedicle length of the free tissue flap of Group A was 11.75 ± 2.30 cm, while in Group B the vascular pedicle length of the free tissue flap was 10.67 ± 2.13 cm (*p* = 0.216). The characteristics of the technical aspects and complications of free flaps in both groups are listed in Table 2.

**Table 2:** Characteristics of the technical aspects and complications of free tissue flaps in both groups.

	Group A	Group B	
	Superficial temporal recipient vessels (n = 12)	Cervical recipient vessels (n = 15)	*p*-value
Size of the free tissue flap (cm^2^)	139 (SD ± 81.45)	178 (SD ± 49.47)	0.206
Vascular pedicle length of the free tissue flap (cm)	11.75 ± 2.30	10.67 ± 2.13	0.216
Harvesting time of the free tissue flap (min.)	137.5 (min. 90 – max. 160)	145 (min. 55 – max. 165)	0.638
Ischemia time	60 (min. 45 – max. 107)	62 (min. 42 – max. 128)	0.971
Major complications of the free tissue flap (total flap loss)	02	01	0.569
Minor complications of the free tissue flap	01	04	0.342
Total surgical time (min.)	621.8 (SD ± 130.7)	646.5 (SD ± 63.03)	0.594

## Discussion

Few articles have specifically compared the postoperative results based on the choice of the recipient vessels in midface and scalp microvascular reconstruction.^12^, ^13^, ^14^ One of the most important factors for the selection of the recipient vessels is the distance from the flap inset location. In our study, the distance among the advanced oncologic defects and the recipient vessels was not significant, probably because we selected free tissue flaps with long vascular pedicle as anterolateral thigh flaps, *latissimus dorsi* myocutaneous flaps, *rectus abdominis* flaps and forearm flaps.^5^, ^6^, ^7^ The average length of the main pedicle of our series was nearly 11 cm, considered adequate tension-free safe anastomosis using either the superficial temporal or the cervical vessels. Anatomically, the superficial temporal vessels are substantially closer to the midface and scalp region than the cervical vessels. Theoretically, this proximity could facilitate the reconstruction, with no need for a vein graft. In fact, in the present series, all microanastomosis were performed primarily, and no vein graft was necessary. We believe that this option decreases time of surgery and reduce the rates of vascular complications associated with vein grafts.

The superficial temporal vessels are readily available, and their suitability as recipient vessels for microvascular free tissue flap in head and neck reconstruction has been reported by numerous authors.^14^, ^15^, ^16^ The major drawback to their use is the tortuous nature of the vessels in this location, their perceived tendency to vasospasm, given their superficial nature, and the concern for proximity to the facial nerve. To improve results and minimize these vascular complications, some authors have describe a dissection even more proximal into the intraparotid segment of the superficial temporal vessel.^17^ Another consideration is that the inset location or positioning, as well as the ultimate reconstructive results, could potentially be influenced by limitations of the vascular pedicle. When using the superficial temporal vessels, however, the length of the vascular pedicle does not need to be nearly as long. This, in turn, makes it easier to tailor the reconstruction of the composite 3-dimensional defects of the midface and scalp.

Although there was no significant difference in the rate of total flap loss between the 2 groups in our series, the rate of complications was nevertheless higher in patients with superficial temporal recipient vessels than in those with cervical recipient vessels. It is important to emphasize that, of the 4 patients who had microvascular anastomosis complications, three were undergoing secondary free tissue flap reconstruction, due to previous total flap loss. In our series, the overall flap survival rate was 88.89%. These data are similar to other available series reporting above 90% of success in free flaps for advanced cases.^7^, ^18^ In cases in whom a secondary reconstruction is performed, cervical vessels are often inadequate or absent; moreover, they are frequently encased in scar tissue due to previous neck dissection or radiotherapy. In this situation, the superficial temporal vessels are probably a better choice, not only because they are anatomically closer, but rather they have not been affected by any previous treatment. In our series, only 4 patients underwent secondary reconstruction; all individuals had already been submitted to either radiotherapy and/or neck dissection. Despite an additional technical difficulty at the cervical preoperative site, this finding did not preclude the performance of a successful microanastomosis.

Wang et al.^19^ reviewed the advantages and disadvantages of using free flaps for anterior skull base defects using both superficial temporal and cervical recipient vessels. They concluded that, depending on the extent of the resection and the length of the vascular pedicle of the free flap, both options are reasonable. Our study demonstrated that both cervical and superficial temporal recipient’s vessels proved to be viable options for the microvascular reconstruction of extensive midface and anterior skull base oncologic defects. We agree with Yazar^20^ that the superficial temporal vessels should be the preferred recipient vessels for microvascular anastomosis in secondary free flap reconstruction, as they are usually located far from the previous surgical dissection. Furthermore, Imanishi et al.^21^ showed that the superficial temporal vein has a complex pattern with numerous branches and connections. These factors make the superficial temporal vein a safe option for dissection and anastomosis, even if the ipsilateral internal jugular vein is ligated. Hansen et al.^22^ described their successful experience with the superficial temporal vessels as recipient vessels for facial and scalp reconstruction.

Our study is not without limitations. First, selection bias could have been an issue, since the main author did patient allocation. Even though, we cannot completely exclude this systematic error, our statistical results indicate that groups were similar in each and every domain, so minimizing the effect of patient selection. Finally, sample size is not expressive, but proved adequate on statistical analysis. It is worth considering the rarity of such advanced cancers of the midface and scalp, even in reference centers, like ours, resulting in very large surgical defects and demanding very complex reconstructions. Our results should be validated in large multicenter studies. On the other hand, our study is the first randomized study in the field providing further support for the use of superficial temporal vessels in such difficult cases of midface and scalp advanced oncologic reconstruction.

## Conclusion

Midface and scalp advanced oncologic reconstruction using cervical or superficial temporal recipient vessels did not differ in terms of success and complication rates. Therefore, the use of superficial temporal recipient vessels may be a reasonable option, especially in a secondary reconstruction setting.

## Funding

The study had own financing.

## Conflicts of interest

The authors declare no conflicts of interest.
