# Supplementary material for: Recipient vessels for free flaps in advanced facial oncologic defects
Source: Braz J Otorhinolaryngol. 2023 Apr 6;89(4):101271. doi: 10.1016/j.bjorl.2023.03.008 (PMC10300290; doi:10.1016/j.bjorl.2023.03.008)
Supplement: Supplementary file 1 [file mmc1.pdf]

We're building a better ClinicalTrials.gov. Check it out and tell us what you think!

U.S. National Library of Medicine
ClinicalTrials.gov

Recipient Vessels for Free Tissue Flaps in Advanced Oncologic Defects of the Midface and Scalp

The safety and scientific validity of this study is the responsibility of the study sponsor and investigators. Listing a study does not mean it has been evaluated by the U.S. Federal Government. Read our disclaimer for details.

ClinicalTrials.gov Identifier: NCT05749120

Recruitment Status : Completed
First Posted : March 1, 2023
Last Update Posted : March 1, 2023

View this study on Beta.ClinicalTrials.gov

Sponsor:
Instituto Nacional de Cancer, Brazil

Information provided by (Responsible Party):
Bruno Albuquerque, MD, Instituto Nacional de Cancer, Brazil

**Tracking Information**
**First Submitted Date** ICMJE
February 18, 2023

First Posted Date ICMJE

March 1, 2023

Last Update Posted Date

March 1, 2023

Actual Study Start Date ICMJE

April 1, 2018

Actual Primary Completion Date

April 30, 2022 (Final data collection date for primary outcome measure)

Current Primary Outcome Measures ICMJE
(submitted: February 18, 2023)

To compare the overall flap survival rate after the microanastomosis [Time Frame: Participants were assessed twice a day for the first 10 days after the intervention and then weekly for 4 weeks.]

Adequate perfusion of the free tissue flap was assessed using Doppler flowmetry, flap bleeding time after puncture, color, texture and turgor. Both venous and arterial thrombosis were evaluated by these methods in order to detect whether there was any evidence of low or absence of vascular perfusion of the flap.

Original Primary Outcome Measures ICMJE

Same as current

Change History

No Changes Posted

Current Secondary Outcome Measures ICMJE

Not Provided

Original Secondary Outcome Measures ICMJE

Not Provided

Current Other Pre-specified Outcome Measures

Not Provided

Original Other Pre-specified Outcome Measures

Not Provided

Descriptive Information

**Brief Title** ICMJE
Recipient Vessels for Free Tissue Flaps in Advanced Oncologic Defects of the Midface and Scalp
**Official Title** ICMJE
Recipient Vessels for Free Tissue Flaps in Advanced Oncologic Defects of the Midface and Scalp: Prospective Randomized Study
**Brief Summary**
The goal of this clinical trial is to compare the postoperative outcomes based on superficial temporal versus cervical recipient vessels for midface and scalp advanced oncologic defects using free tissue flap for reconstruction.<br>The main question it aims to answer is:<br>• Which recipient vessel is most suitable for performing microanastomosis using free flaps for advanced midface and scalp oncologic defect.<br>Participants will be undergo resection of advanced malignant tumors of the midface and scalp with subsequent oncological reconstruction using free tissue flap.<br>Researchers will compare two groups where those in whom superficial temporal vessels will be used as the recipient vessels (group A) and those in whom cervical vessels will be used as the recipient vessels (group B) to see if there is a recipient vessel who is most suitable for performing microanastomosis using free flaps for advanced midface and scalp oncologic defect.
**Detailed Description**
Advanced oncologic defects of the midface and scalp are a significant challenge to the reconstructive head neck surgeon, who must consider the need for midfacial projection, rehabilitation, and function restoration. Free flaps reconstruction in the midface and scalp region are the gold standard for advanced cases. There is no consensus in the literature on which recipient vessel is most suitable for performing microanastomosis using free flaps for advanced midface and scalp oncologic defect. The aim of this clinical trial is prospectively compare the results of microvascular flap reconstruction of midface and scalp advanced oncologic defects using superficial temporal versus cervical as recipient vessels. This is a parallel trial with permuted block randomization of patients who will be undergone a midface and scalp oncologic reconstruction with free tissue flap. Two groups will be analyzed: those in whom superficial temporal vessels will be used as the recipient vessels (group A) and those in whom cervical vessels will be used as the recipient vessels (group B). Allocation ratio will de 1:1 participants. Patient gender and age, cause and localization of the defect, flap choice for reconstruction, recipient vessels, intraoperative outcome, postoperative course, and complications were recorded and analyzed. Considering that this is a disease with rare staging, the calculated sample size was 26, but it will be increased by 30% (total of 34 participants who will be selected) since the death rate of these patients ranges from 8-25% A Fisher's exact test will be used to compare outcomes between the 2 groups.
**Study Type** ICMJE
Interventional
**Study Phase** ICMJE

Not Applicable

Study Design ICMJE

Allocation: Randomized
Intervention Model: Parallel Assignment
Intervention Model Description:
Parallel group trial design with allocation ratio of 1:1 and permuted block randomization
Masking: Single (Participant)
Masking Description:
The care provider knows the surgical intervention and the randomized participant doesn´t know the recipient vessel performed
Primary Purpose: Treatment

Condition ICMJE

Head Neck Neoplasms

Intervention ICMJE

Procedure: Recipient vessel selected for microanastomosis
Free tissue flaps harvesting and microanastomosis were performed by the same surgeon and in a similar way in both group. Postoperative course, and complications were recorded and analyzed.

Study Arms ICMJE

- Experimental: Superficial temporal recipient vessels (group A)
  Participants in whom superficial temporal vessels were used as the recipient vessels.
  Intervention: Procedure: Recipient vessel selected for microanastomosis
- Active Comparator: Cervical recipient vessels (group B)
  Participants in whom cervical vessels were used as the recipient vessels.
  Intervention: Procedure: Recipient vessel selected for microanastomosis

Publications *

- Awwad L, Obed D, Vogt PM, Kaltenborn A, Koenneker S. Superficial Temporal Recipient Vessels for Craniofacial Microvascular Free-Flaps. J Craniofac Surg. 2022 Sep 1;33(6):e652-e657. doi: 10.1097/SCS.0000000000008768. Epub 2022 Jul 22.
- Mata Ribeiro L, Tsao CK, Hung YL, Chu CH, Lin LC, Lin MH, Peng C, Cheong DC, Hung SY, Liao CT. Venous Size Discrepancy Is a Critical Factor When Using Superficial Temporal Vessels as Recipient Vessels for Free Flaps. J Reconstr Microsurg. 2022 Oct;38(8):654-663. doi: 10.1055/s-0042-1743165. Epub 2022 Feb 25.
- Sudirman SR, Shih HS, Chen JC, Feng KM, Jeng SF. Superficial temporal vessels, both anterograde and retrograde limbs, are viable recipient vessels for recurrent head and neck

reconstruction in patients with frozen neck. Head Neck. 2019 Oct;41(10):3618-3623. doi: 10.1002/hed.25886. Epub 2019 Jul 26.

- Mattine S, Payne KFB. The evolving role of the superficial temporal vessels as anastomotic recipients in challenging microvascular reconstruction of the upper two-thirds of the face. J Plast Reconstr Aesthet Surg. 2022 Sep;75(9):3330-3339. doi: 10.1016/j.bjps.2022.04.089. Epub 2022 May 2.

*** Includes publications given by the data provider as well as publications identified by ClinicalTrials.gov Identifier (NCT Number) in Medline.**

Recruitment Information

Recruitment Status ICMJE

Completed

Actual Enrollment ICMJE
(submitted: February 18, 2023)

32

Original Actual Enrollment ICMJE

Same as current

Actual Study Completion Date ICMJE

June 30, 2022

Actual Primary Completion Date

April 30, 2022 (Final data collection date for primary outcome measure)

Eligibility Criteria ICMJE

Inclusion Criteria:

- Patients submitted to subtotal, total, or extensive radical oncologic maxillectomy, or advanced scalp oncologic defect
- Complete medical records for at least 30 days after the intervention
- Patients eligible for microanastomosis in both cervical and superficial temporal recipient vessels

Exclusion Criteria:

- Pregnancy or breastfeeding;
- Serious diseases (sepsis, renal and hepatic failure, severe cardiovascular diseases, chronic obstructive pulmonary disease);

- Patients with HIV (human immunodeficiency virus) with or without AIDS (acquired immunodeficiency syndrome);
- Patients with synchronous malignant neoplasms of the upper airway-digestive tract;
- Patients who died within a period of less than 30 days or who were lost to follow-up for the same period

Sex/Gender ICMJE

Sexes Eligible for Study:
All

Ages ICMJE

14 Years and older (Child, Adult, Older Adult)

Accepts Healthy Volunteers ICMJE

No

Contacts ICMJE

Contact information is only displayed when the study is recruiting subjects

Listed Location Countries ICMJE

Brazil

Removed Location Countries

Administrative Information

NCT Number ICMJE

NCT05749120

Other Study ID Numbers ICMJE

89042418.7.0000.5274

Has Data Monitoring Committee

Yes

U.S. FDA-regulated Product

Studies a U.S. FDA-regulated Drug Product:
No

Studies a U.S. FDA-regulated Device Product:
No

**IPD Sharing Statement** ICMJE	**Plan to Share IPD:** No
**Current Responsible Party**	Bruno Albuquerque, MD, Instituto Nacional de Cancer, Brazil
**Original Responsible Party**	*Same as current*
**Current Study Sponsor** ICMJE	Instituto Nacional de Cancer, Brazil
**Original Study Sponsor** ICMJE	*Same as current*
**Collaborators** ICMJE	*Not Provided*
**Investigators** ICMJE	**Principal Investigator:** Bruno A Sousa, MD, Brazilian National Cancer Institute
**PRS Account**	Instituto Nacional de Cancer, Brazil
**Verification Date**	February 2023

ICMJE **Data element required by the International Committee of Medical Journal Editors and the World Health Organization ICTRP**
